# Safety and convenience of once-weekly somapacitan in adult GH deficiency: a 26-week randomized, controlled trial

**DOI:** 10.1530/EJE-17-1073

**Published:** 2018-02-26

**Authors:** Gudmundur Johannsson, Ulla Feldt-Rasmussen, Ida Holme Håkonsson, Henrik Biering, Patrice Rodien, Shigeyuki Tahara, Andrew Toogood, Michael Højby Rasmussen, the REAL 2 Study Group, the REAL 2 Study Group, Henrik Biering, Wolfram Karges, Alexander Mann, Jens Sandahl Christiansen, Troels Krarup Hansen, Marianne Andersen, Ulla Feldt-Rasmussen, Sine Borresen, Patrice Rodien, Françoise Borson-Chazot, Véronique Kerlan, Bertrand Cariou, Bruno Verges, Shigeyuki Tahara, Akira Matsuno, Koji Takano, Tetsuya Tagami, Yutaka Takahashi, Toshikazu Takahashi, Masahiro Yamamoto, Gudmundur Johannsson, Charlotte Höybye, Eva-Marie Erfurth, William Drake, Claire Higham, Robert Murray, Andrew Toogood, Antonia Brooke

**Affiliations:** 1 University of Göteborg and Sahlgrenska University HospitalGöteborg, Sweden; 2 RigshospitaletCopenhagen, Denmark; 3 Global DevelopmentNovo Nordisk A/S, Søborg, Denmark; 4 MediCover Berlin-Mitte MVZBerlin, Germany; 5 Reference Centre for Rare Diseases of Thyroid and Hormone ReceptorsMember of EndoERN Network, CHU Angers Centre Hospitalier Universitaire, Angers, France; 6 Nippon Medical SchoolTokyo, Japan; 7 Queen Elizabeth Hospital BirminghamBirmingham, UK

## Abstract

**Objective:**

Somapacitan is a reversible albumin-binding growth hormone (GH) derivative, developed for once-weekly administration. This study aimed to evaluate the safety of once-weekly somapacitan vs once-daily Norditropin^®^. Local tolerability and treatment satisfaction were also assessed.

**Design:**

26-week randomized, controlled phase 3 safety and tolerability trial in six countries (Nbib2382939).

**Methods:**

Male or female patients aged 18–79 years with adult GH deficiency (AGHD), treated with once-daily GH for ≥6 months, were randomized to once-weekly somapacitan (*n* = 61) or once-daily Norditropin (*n* = 31) administered subcutaneously by pen. Both treatments were dose titrated for 8 weeks to achieve insulin-like growth factor I (IGF-I) standard deviation score (SDS) levels within the normal range, and then administered at a fixed dose. Outcome measures were adverse events (AEs), including injection site reactions; occurrence of anti-somapacitan/anti-GH antibodies and change in treatment satisfaction, assessed using the Treatment Satisfaction Questionnaire for Medication-9 (TSQM-9).

**Results:**

Mean IGF-I SDS remained between 0 and 2 SDS throughout the trial in both groups. AEs were mostly mild or moderate and transient in nature. The most common AEs were nasopharyngitis, headache and fatigue in both groups. More than 1500 somapacitan injections were administered and no clinically significant injection site reactions were reported. No anti-somapacitan or anti-GH antibodies were detected. The TSQM-9 score for convenience increased significantly more with somapacitan vs Norditropin (*P* = 0.0171).

**Conclusions:**

In this 26-week trial in patients with AGHD, somapacitan was well tolerated and no safety issues were identified. Once-weekly somapacitan was reported to be more convenient than once-daily Norditropin.

## Introduction

Adult growth hormone deficiency (AGHD) is characterized by several clinical features that compromise general health and quality of life. If left untreated, AGHD is associated with disturbed lipoprotein metabolism, reduced exercise capacity, increased body fat, reduced bone mineral density (BMD), increased risk of cardiovascular morbidity and mortality and decreased cognition and psychological well-being (1, 2, 3, 4). Treatment of AGHD aims to prevent or ameliorate the long-term complications of growth hormone deficiency (GHD) and improve quality of life (QoL) (1, 3). Human GH replacement has been shown to be an effective treatment with regard to these aims, with a favorable safety profile, both in randomized studies against placebo (reviewed in (2, 5)) and in longer-term open studies and studies based on records from surveillance databases (6, 7).

GH replacement is generally administered by daily subcutaneous injections, resulting in a pharmacokinetic profile far from the physiological pulsatile pattern of endogenous GH secretion. Nevertheless, the safety and efficacy of daily GH injections for the treatment of adults and children with GHD have been confirmed in numerous clinical studies (2). Although daily GH is administered with the use of fine needles which minimize pain, some patients still find a daily regimen burdensome, particularly because treatment may last for several years or even be lifelong. It is well established that poor adherence with medication is associated with poorer clinical outcomes (8). Some studies of patients with AGHD have reported poor adherence rates – for example, a survey of 158 adult patients who were receiving or had received GH therapy rated only 34% as ‘highly compliant’ (9), and a recent retrospective single-center cohort study classified adherence as <20% in 6.6% of 179 patients with AGHD (10). A reduction in the frequency of injections could potentially improve adherence and thus clinical outcomes.

Somapacitan (Novo Nordisk A/S) is a novel reversible albumin-binding GH derivative in which fatty acids with non-covalent albumin-binding properties have been conjugated by alkylation to GH. The resulting non-covalent binding of the GH molecule to endogenous albumin reduces the clearance and extends the half-life of the drug. Pharmacokinetic studies in healthy adults (11) and both children (12) and adults with GH deficiency (13) showed that the concentration of somapacitan peaked at 1–2 days after injection and returned to low levels after 7 days, and pharmacodynamic studies showed C_max_ for insulin-like growth factor I (IGF-I) and IGF-I SDS4 days after dosing, with values subsequently falling gradually, supporting the possibility of once-weekly subcutaneous administration. A similar technology of using a conjugated linker to extend the plasma half-life of a peptide drug has previously been used successfully in the development of insulin detemir, a long-acting insulin analog (14), and liraglutide, a long-acting glucagon-like peptide-1 (GLP-1) derivative (15) without any associated significant tolerability issues. Despite a similar protraction technology being applied, somapacitan has a different receptor pharmacology and different albumin-binding properties, resulting in a longer residual time in circulation compared with insulin detemir and liraglutide. This, together with the stimulation of the IGF-I secretion, makes somapacitan active for a longer interval.

Somapacitan was also shown in short-term trials to be well tolerated in healthy adults (11), and both children (12) and adults with GH deficiency (13).We now report the first data obtained from a trial of somapacitan investigating the clinical safety and tolerability of, and treatment satisfaction with, once-weekly somapacitan vs once-daily GH (Norditropin^®^ FlexPro^®^, Novo Nordisk A/S) over 26 weeks in patients with AGHD previously treated with daily GH.

## Subjects and methods

### Patients

Male or female patients aged 18–79 years diagnosed with AGHD and treated with once-daily GH for ≥6 months were eligible for the trial. The diagnosis of AGHD could include GHD of either adult onset (diagnosed alone or associated with multiple hormone deficiencies) or childhood onset. The diagnosis of AGHD was made in accordance with the GH Research Society guidelines (16), Endocrine Society guidelines (1) or relevant guidelines applicable at the time of AGHD diagnosis.

Full inclusion and exclusion criteria are shown in Supplementary Table 1 (see section on supplementary data given at the end of this article).

### Trial design and procedures

This was a multinational, multicenter, randomized, open-label, active-controlled trial (ClinicalTrials.gov: Nbib2382939; REAL 2), conducted at 26 sites in six countries between February 2015 and January 2016. The protocol was approved by the local and national ethics committees, as appropriate and conducted in accordance with the International Conference on Harmonisation guidelines for Good Clinical Practice (International Conference on Harmonisation. Harmonised Tripartite Guideline for Good Clinical Practice. Geneva, Switzerland. 1996. http://www.ich.org/products/guidelines/efficacy/efficacy-single/article/good-clinical-practice.html (accessed 18 July 2017)) and the Declaration of Helsinki (WMA. Declaration of Helsinki: Ethical principles for medical research involving human subjects. Last amended by the 64th WMA General Assembly (Brazil). Oct 2013. https://www.wma.net/policies-post/wma-declaration-of-helsinki-ethical-principles-for-medical-research-involving-human-subjects/ (accessed 18 July 2017)). Informed consent was obtained from all patients prior to inclusion.

Patients were randomized 2:1 to receive once-weekly somapacitan or once-daily Norditropin (Supplementary Fig. 1). The randomization process is described in the Supplementary material. Somapacitan was administered at a starting dose of 1.0−1.5 mg/week (2.0 mg/week for women on oral estrogen treatment), and Norditropin at a starting dose of 0.1−0.2 mg/day (0.3 mg/day for women on oral estrogen treatment). Both treatments were administered via subcutaneous injection: somapacitan was administered in the morning and Norditropin was administered in the evening, both using a prefilled FlexPro pen.

During the first 8 weeks, somapacitan and Norditropin doses were titrated according to serum IGF-I standard deviation score (SDS) in order to achieve IGF-I levels within the normal range, and preferably between 0 and 2 SDS. A fixed dose was administered for the remaining 18-week period. Sampling of IGF-I during the titration phase was planned at Day 3–4, where the IGF-I value was expected to represent the average IGF-I value during the week. Maximum recommended doses were somapacitan 8.0 mg/week or Norditropin 1.1 mg/day (1.0 mg/day in Japan). Somapacitan doses of greater than 4 mg were split into two injections of equal volume (required for nine patients).

Two washout periods were included for the purpose of measuring antibodies: a 1-day washout before randomization and a 1-week washout after Week 26 (Supplementary Fig. 1).

During the trial period, patients were instructed by the site staff to record dose adherence (date and time of each dose of trial product as well as any missed dose). Adherence (%) was calculated as the number of reported doses from patient diaries and doses administered during clinic visits, divided by number of planned doses, multiplied by 100.

### Safety assessments

Safety was assessed in terms of the incidence of AEs, including injection site reactions, from baseline to the end of the post-treatment follow-up period (1 week after end of treatment). All AEs either observed by the investigator, reported spontaneously by the patients, or reported in response to questioning at each site visit, were recorded and evaluated. Injection site reactions were evaluated at each visit by manual, visual inspection of injection sites and assessment of the occurrence of pain, tenderness, itching, rash, redness, induration and any other signs of injection site reactions. Injection site reactions could also be reported by the patient between visits.

Other safety assessments included physical examination, body weight, vital signs, electrocardiograms, clinical laboratory tests (hematology, biochemistry, urinalysis, fasting blood glucose and fasting blood insulin) and the occurrence of anti-somapacitan or anti-GH antibodies. Samples for glucose and insulin were taken on the same day as dosing with somapacitan (Week 8) or 4 days after dosing with somapacitan (Weeks 16 and 25).

### Treatment satisfaction

The change in treatment satisfaction from randomization to Week 26 was assessed using the Treatment Satisfaction Questionnaire for Medication-9 (TSQM-9) (17). The original TSQM-14 was shown to be a psychometrically sound and valid measure of the major dimensions of satisfaction with medication in patients with different chronic diseases (18). An abbreviated version that omitted questions on side effects, the TSQM-9, was subsequently also validated (17). Items on the TSQM-9 are rated on a 5- or 7-point scale, with an increase in scores signifying an increase in treatment satisfaction and can be grouped to provide effectiveness, convenience and global satisfaction scores. Questionnaires were completed by the patients, without assistance from site personnel, at randomization and at Weeks 16 and 26.

### Assay methods

Analysis of serum IGF-I and insulin-like growth factor-binding protein-3 (IGFBP-3) concentration was performed using commercially available assay kits (Immuno Diagnostic Systems immunoassay ISYS assay at the analytical central laboratory PPD Global Central Labs, BVBA, Zaventem, Belgium). Somapacitan was dosed on Day 1 of each week and samples for serum IGF-I analysis were taken on Day 4, except for Week 4, when samples were taken on Day 7 in order to establish the trough level. IGF-I and IGFBP-3 assay performance was in accordance with the assay information provided by the manufacturer. The IGF-I assay was calibrated using World Health Organization International Standard 02/254 and IGF-I SDSs were calculated according to Bidlingmaier *et al*. (19).

Anti-somapacitan and anti-GH antibodies were also assessed, using validated bridging ELISAs developed by Novo Nordisk to specifically determine antibody levels against somapacitan and human GH, respectively (for details see (13)).

### Statistical analysis

The sample size was not based on any formal calculations apart from accounting for withdrawals in the final sample size. Based on, at most, 15% withdrawals per treatment arm, it was estimated that 90 patients should be randomized in a 2:1 ratio between somapacitan and Norditropin. The 15% withdrawal rate was considered conservative and acceptable relative to other trials with similar trial designs, in particular considering that this trial had no placebo arm and did not include any invasive or unpleasant investigations.

The safety analysis and full analysis sets both included all randomized subjects who received at least one dose of treatment. The primary endpoint and all safety endpoints were reported by descriptive statistics.

Estimated treatment differences in TSQM-9 effectiveness, convenience and global satisfaction scores at 26 weeks were estimated from a mixed model for repeated measurements, with treatment, GHD onset type, sex, region and sex by region interaction term as factors and baseline as a covariate, all nested within week as a factor. Patients without post-randomization data for the analyzed endpoint were not included in the analysis.

## Results

### Patient disposition

Of 98 screened patients, 92 were randomized to receive once-weekly somapacitan (*n* = 61) or once-daily Norditropin (*n* = 31). Characteristics of the randomized patients were well matched at baseline (Table 1). All 92 AGHD patients were included in the full analysis set and the safety analysis set, which were identical.

**Table 1:** Baseline characteristics.

	Somapacitan once-weekly (*n* = 61)	Norditropin once-daily (*n* = 31)
Age (years), mean (s.d.)	48.1 (16.2)	51.7 (17.1)
Female, *n* (%)	28 (45.9)	14 (45.2)
Race, *n* (%)		
Asian	12 (19.7)	6 (19.4)
White	36 (59.0)	18 (58.1)
Not available	13 (21.3)	7 (22.6)
Body weight (kg), mean (s.d.)	82.1 (17.6)	81.0 (21.8)
BMI (kg/m^2^), mean (s.d.)	28.6 (5.0)	28.5 (5.6)
GHD onset, *n* (%)		
Childhood – idiopathic	6 (9.8)	3 (9.7)
Childhood – organic	18 (29.5)	7 (22.6)
Adulthood	37 (60.7)	21 (67.7)
IGF-I SDS	0.28 (1.50)	0.91 (1.24)
GH dose level at screening (mg), mean (s.d.)	0.5 (0.3)	0.5 (0.9)

BMI, body mass index; GH, grouwth hormone; GHD, growth hormone deficiency; SDS, standard deviation score.

All randomized patients were exposed to their assigned treatment, and 86 patients completed the trial. Three patients in each treatment group withdrew (somapacitan: 4.9%; Norditropin: 9.7%). In each arm, two patients withdrew consent to participate in the trial, and one patient withdrew due to AEs as follows: for somapacitan: asthenia and disturbance in attention; and for Norditropin, asthenia, somnolence, disturbance in attention and headache. One participant discontinued somapacitan treatment due to travel abroad without withdrawing from the trial.

### Dosing

After the dose titration period, the mean somapacitan dose (SD) was 1.96 (1.45) mg/week in the 18 weeks of fixed-dose treatment, compared with a starting dose of 1.5 mg/week. For Norditropin, the mean dose after titration was 0.20 (0.14) mg/day, which was similar to the starting dose.

The mean days of exposure were similar in the two groups: 177 (range: 7–196) days for somapacitan and 172 (10–184) days for Norditropin.

Mean treatment adherence by patients during the trial, measured as defined previously under Trial Design and Procedures (i.e. percentage of doses taken correctly), was 93.1% (range: 3.8–100.0%) in the somapacitan group and 90.4% (5.6–100.0%) in the Norditropin group.

### IGF-I levels

**Figure 1:**
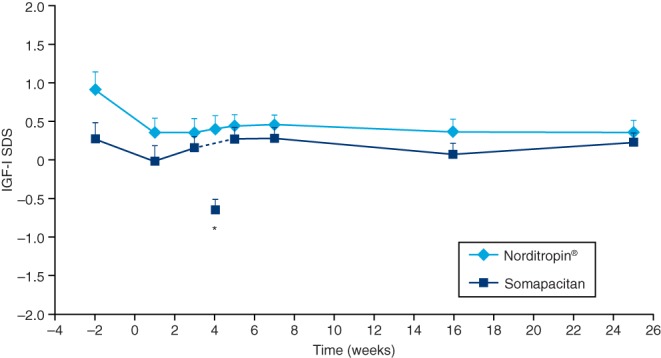
Serum IGF-I SDS levels (mean + s.e.m.) vs time. *Week 4 is the trough value, measured before administration of somapacitan. SDS, standard deviation score.

Mean IGF-I SDS values were maintained throughout the trial, remaining between 0 and 2 SDS in both treatment groups. At the end of trial, IGF-I SDS values were similar in the two groups: mean (s.d.) 0.22 (0.89) for somapacitan and 0.35 (0.82) for Norditropin (Fig. 1). No IGF-I SDS mean values were above +2 in the fixed-dose treatment period. The initially wide range in IGF-I SDS scores tended to decrease during the trial (somapacitan: range from –4.23; 2.80 to –2.73; 1.65; Norditropin: range from –1.63; 3.10 to –1.51; 1.81). Individual IGF-I SDS values above +2 were observed only in the titration period (somapacitan: seven of 61 patients; Norditropin: four of 31 patients).

### Safety

#### AEs and serious AEs (SAEs)

AEs were reported by 86.9% and 67.7% of patients in the somapacitan and Norditropin groups, respectively. Rates of AEs per 100 patient-years of exposure were 514.2 and 530.8, respectively (Table 2). One patient in each treatment group withdrew due to AEs.

**Table 2:** Adverse events.

	Somapacitan once-weekly (*n* = 61)			Norditropin once-daily (*n* = 31)		
	*n* (%)	E	Rate	*n* (%)	E	Rate
Adverse events	53 (86.9)	159	514.2	21 (67.7)	81	530.8
Serious adverse events	4 (6.6)	4	12.9	2 (6.5)	3	19.7
Severity						
Mild	44 (72.1)	119	384.8	18 (58.1)	58	380.1
Moderate	16 (26.2)	32	103.5	8 (25.8)	19	124.5
Severe	5 (8.2)	8	25.9	2 (6.5)	4	26.2
Relationship to trial drug						
Unlikely related	46 (75.4)	130	420.4	21 (67.7)	68	445.6
Possibly related	9 (14.8)	15	48.5	3 (9.7)	5	32.8
Probably related	8 (13.1)	14	45.3	4 (12.9)	8	52.4

E, number of adverse events; *n*, number of patients with adverse events; Rate, adverse event rate/100 patient-years.

The reported AEs were similar overall to AEs observed in previous trials with daily human GH (hGH) treatment, with nasopharyngitis, headache and fatigue as the most frequently occurring AEs for both somapacitan and Norditropin (Table 3); no cases of headache were related to intracranial hypertension as assessed by clinical investigators. The majority of AEs were single events reported in one or two patients and of mild/moderate severity. The most frequent AEs were reported with a greater frequency and event rate in the Norditropin group than in the somapacitan group. There were no reports of peripheral edema during treatment. Carpel tunnel syndrome was reported as a concomitant illness in one patient at baseline.

**Table 3:** Adverse events occurring in ≥5% of patients in either treatment group.

	Somapacitan		Norditropin	
	%	Rate	%	Rate
Nasopharyngitis	19.7	42.0	25.8	72.1
Headache	11.5	35.6	19.4	65.5
Fatigue	9.8	22.6	16.1	32.8
Dizziness	1.6	3.2	9.7	19.7
Arthralgia	8.2	16.2	6.5	13.1
Abdominal pain	6.6	12.9	0.0	0.0
Asthenia	6.6	16.2	3.2	6.6
Sciatica	6.6	12.9	0.0	0.0
Depression	0.0	0.0	6.5	13.1
Gamma-glutamyltransferase increased	0.0	0.0	6.5	13.1
Central hypothyroidism (secondary)	1.6	3.2	6.5	13.1

Rate, event rate/100 patient-years.

SAEs (*n* = 7) were reported as follows: in the somapacitan group, cholelithiasis, procedural complication, mammoplasty and patella fracture (all *n* = 1 event in one patient each); and in the Norditropin group, intestinal ischemia and short-bowel syndrome (*n* = 2 events, both in one patient) and nephrolithiasis (*n* = 1 event in one patient). The ‘procedural complication’ refers to a patient who withdrew after 1 week in the trial following complications of a pre-planned meniscus operation and was lost to follow-up. All patients recovered from their SAE, except the patient with procedural complications who was lost to follow-up. All SAEs were judged unlikely to be related to trial product by the investigator. There were no changes in individual doses due to the SAEs, and no SAEs that led to withdrawal.

The most frequent AEs assessed as possibly/probably drug related in the somapacitan group were fatigue (five patients), asthenia (three patients) and weight increase (three patients) and in the Norditropin group, fatigue (four patients). Other AEs assessed as possibly/probably drug related were reported in only one or two patients in each group.

No clinically relevant changes were observed upon physical examination or in body weight, vital signs, electrocardiograms or clinical laboratory measurements.

**Figure 2:**
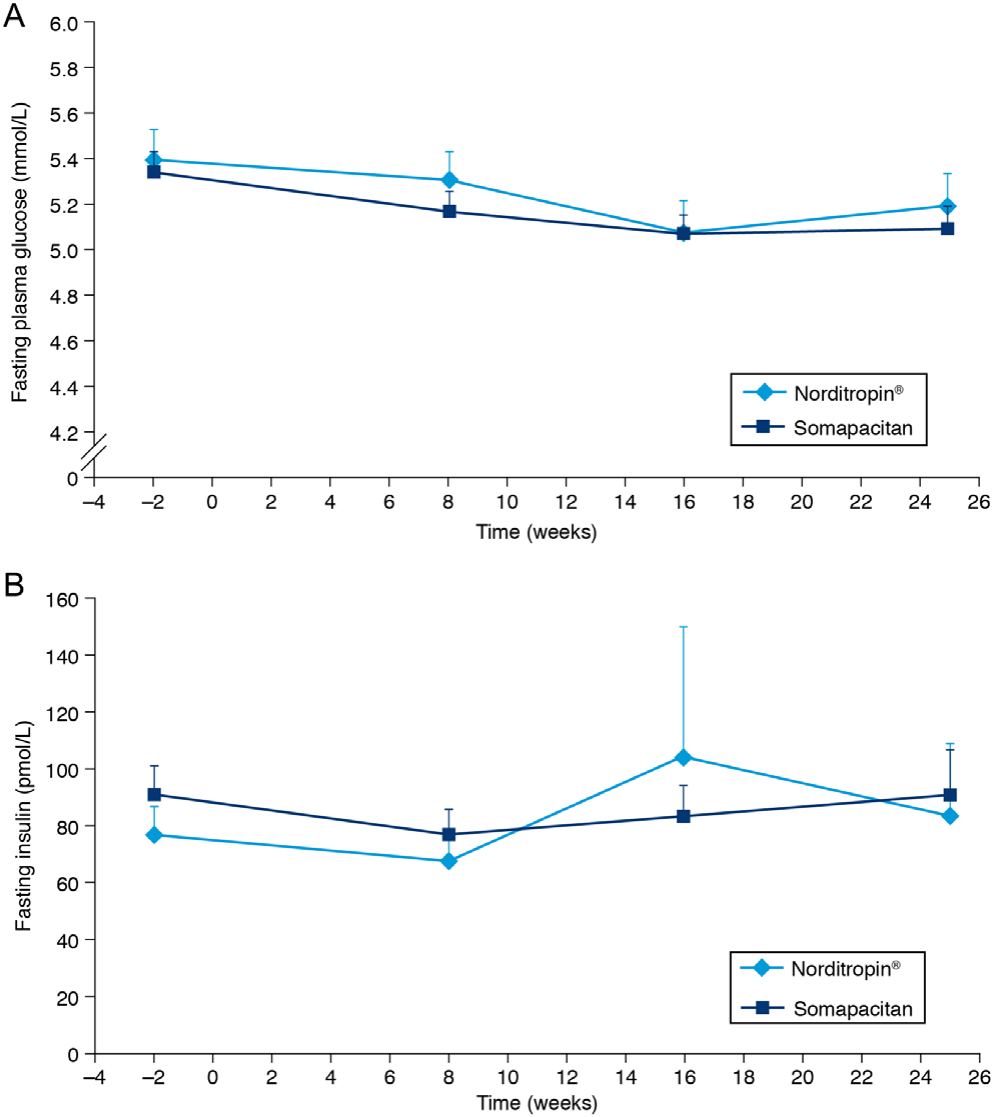
(A) Fasting plasma glucose values and (B) fasting plasma insulin vs time. Values are mean + s.e.m.

Fasting plasma glucose remained stable (Fig. 2A), and no new cases of diabetes were reported during the trial. There were no changes in the mean glycosylated hemoglobin (HbA_1c_), which was similar across the treatment groups. There was some variation in fasting insulin during the trial (Fig. 2B).

#### Local tolerability

The total number of injections in the somapacitan arm was more than 1500. No injection site reactions were reported that were considered to be clinically significant. Two mild and transient injection site reactions were observed in patients treated with somapacitan: hematoma in one patient after the third dose and bruising in one patient after the second dose. No injection site reactions were reported with Norditropin. There were no observations of lipoatrophy or lipohypertrophy in any patients.

#### Antibodies

No anti-somapacitan or anti-GH antibodies were detected.

### Treatment satisfaction

At baseline, the patients’ evaluation of the effectiveness and convenience of the treatment appeared similar in the two treatment arms, and global treatment satisfaction appeared slightly greater in the daily Norditropin arm (not tested for significance) (Supplementary Table 2).

**Figure 3:**
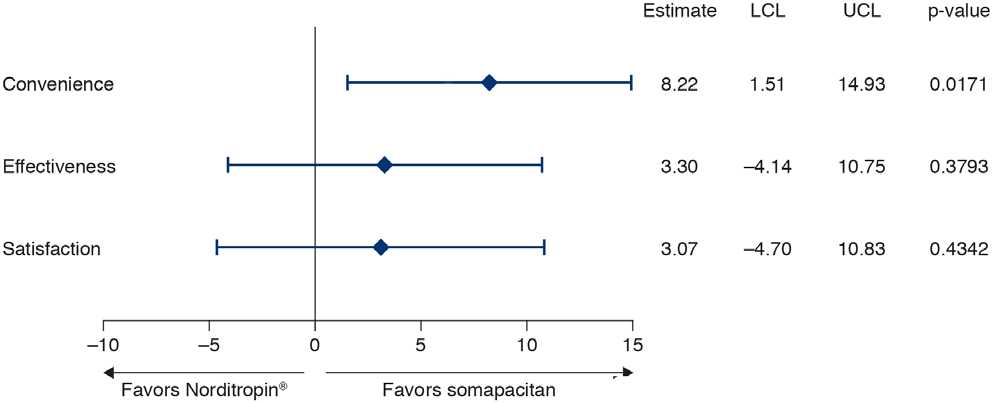
Estimated treatment difference in change in TSQM-9 scores at Week 26. Full analysis set. Estimates are from a mixed model for repeated measurements. LCL, lower confidence limit; TSQM-9, Treatment Satisfaction Questionnaire for Medication-9; UCL, upper confidence limit.

All three categories of TSQM-9 scores (effectiveness, convenience, satisfaction) increased from baseline to Week 26 (Supplementary Table 2). The mean (s.d.) TSQM-9 score for convenience increased by 15.3 (20.9) from 68.3 (18.3) at baseline to 83.8 (12.9) at the end of trial for somapacitan, and by 3.0 (16.5) from 71.7 (17.5) to 75.8 (19.1) with Norditropin. The between-treatment difference at the end of trial was statistically significant (*P* = 0.0171) (Fig. 3). The changes in effectiveness and treatment satisfaction scores did not differ significantly between somapacitan and Norditropin after 26 weeks of treatment (Fig. 3). Thus, after 26 weeks of treatment, once-weekly somapacitan was considered more convenient than daily Norditropin.

## Discussion

In this 26-week randomized study, multiple doses of once-weekly somapacitan were well tolerated; no clinically significant safety or local tolerability issues (including injection site reactions) were reported and the safety profile was very similar to that of daily GH. Dose titration achieved mean IGF-I SDS values of between 0 and 2 SDS in both treatment groups. The evaluation of treatment satisfaction revealed that patients rated once-weekly somapacitan injections as more convenient than once-daily GH injections.

Somapacitan is a novel GH derivative that binds reversibly to endogenous albumin, thus extending its half-life. The safety profile reported in this trial was similar to that observed in previous short-term trials of somapacitan in healthy adults (11) and in adults with AGHD (13). Most events were mild or moderate and transient in nature. Furthermore, the most common AEs, and those judged to be probably/possibly drug-related by the investigator, were AEs that are commonly observed in GH-treated AGHD patients, such as headache, fatigue and asthenia (5). Notably, no clinically significant injection site reactions were reported. This is reassuring because some previous attempts to develop long-acting GH treatments have been hampered by local tolerability problems (20). These have included lipoatrophy with a pegylated formulation (21), nodules and post-injection pain with a sustained-release formulation based on microspheres (22), injection site reactions with a sustained-release formulation (23) and injection site pain in approximately 50% of patients with a GH molecule fused to long-chain amino acids (24).

GH affects insulin sensitivity and may therefore adversely affect glucose metabolism (7, 25); hence, special attention on the effects of GH on glucose metabolism is warranted. In the current study, fasting plasma glucose levels, fasting insulin levels and HbA_1c_ values did not increase markedly over the 26 weeks of the trial, and no cases of diabetes were diagnosed. These results are in line with a recent analysis of real-life data from patients with AGHD, which showed that, in most patients, glucose homeostasis was not adversely affected after 4 years of daily GH treatment (26). It has been reported that insulin resistance is present in adult patients with AGHD and that this is worsened by treatment with GH (25). It has also been suggested that the favorable effects of long-term GH therapy on body composition may counteract the direct insulin antagonistic effects (25).

The IGF-I SDS profile was maintained throughout the trial in both treatment groups, with mean values of between 0 and +2 SDS. IGF-I SDS values >2 were observed in only a few patients, in similar proportions in the somapacitan and Norditropin groups, and only during the titration phase. The decrease in the range of IGF-I SDS values during the trial may reflect the effect of the titration. These results support once-weekly dosing of somapacitan in patients with AGHD and indicate that the dose titration used was safe and efficient for male and female adult patients (with a modification for women on oral estrogen treatment, as described in the Materials and methods section).

In this trial, patient satisfaction was assessed using the TSQM-9. The change in the TSQM-9 convenience score from randomization to Week 26 was statistically significantly greater with somapacitan than with Norditropin. Changes in effectiveness and satisfaction were numerically greater with somapacitan than with Norditropin, but the differences did not reach statistical significance. This finding is in line with reports of patient preference for weekly rather than daily drug administration from other disease areas such as osteoporosis (27) and diabetes (28).

To our knowledge, no published studies are available comparing adherence rates with once-weekly injections vs once-daily injections. In the current trial, adherence with therapy, measured as the percentage of correct doses taken, was high: 93.1% with somapacitan and 90.4% with Norditropin. Adherence was thus very similar between the groups, possibly as a result of the controlled nature of the clinical trial, with regular clinic visits and assessments, as well as highly motivated participating patients. These factors may have contributed to rates of adherence higher than those reported in real life in adults with GHD receiving daily injections (9, 10). We can only speculate that, if the greater convenience of a once-weekly regimen translates into increased adherence with therapy in real-life conditions, this could help maximize the efficacy of long-term GH replacement.

Limitations of this trial are the fact that efficacy was not measured through clinical outcomes, such as changes in body composition and quality of life, as the patients were already receiving long-term stable GH treatment before entering the study. On the other hand, study strengths lie in the fact that it was a randomized trial in which somapacitan was compared head to head with hGH in AGHD patients already using daily GH replacement treatment. Thus, the current study adds important data for patients who will switch from daily to weekly therapy. The patients who were enrolled were well characterized, included both sexes and were recruited from several centers in five different European countries and Japan, thus increasing the generalizability of the results to real-life clinical practice.

In conclusion, in this 26-week trial in patients with AGHD already receiving stable replacement therapy with daily GH, the switch to somapacitan was well tolerated and stable serum IGF-I concentrations within the normal range were achieved. Clinically significant safety issues were not identified, nor were any immunogenicity concerns revealed. In addition, treatment satisfaction responses revealed that the patients considered once-weekly somapacitan injections more convenient than once-daily GH injections and adherence to treatment was high.

## Supplementary Material

Supporting Figure 1

Supplementary material

Supporting Table 1

Supporting Table 2

## Declaration of interest

G J has received speaker’s honoraria from Eli Lilly, Merck Serono, Novartis, Novo Nordisk, Pfizer, Otsuka and Shire and has been a consultant for AstraZeneca, Merck, Serono, Novo Nordisk, Pfizer and Shire. UF-R has received honoraria for teaching from Novo Nordisk, Novartis, Merck, Shire, IPSEN Pharma and Pfizer and is a member of Pfizer’s KIMS Advisory Board. IHH and MHR are employees of Novo Nordisk A/S and own shares in the company. H B has received payments as an investigator in studies for Novo Nordisk and Novartis, as well as for being a consultant for Ipsen. PR has received honoraria for conferences and/or hospitality from Novo Nordisk, IPSEN Pharma, Eli Lilly, Pfizer, Sandoz, Merck Serono; research grants from Sandoz, Pfizer, Novartis and unrestricted educational support from Novo Nordisk. ST and AT have nothing to declare.

## Funding

The trial was financially supported by Novo Nordisk A/S, Denmark. Medical writing and submission support were funded by Novo Nordisk A/S.

## Author contribution statement

All authors confirm that they made a substantial contribution to research design, or the acquisition, analysis or interpretation of data; have revised the manuscript critically and have approved the final version.
