# Supplementary figures and images for: Safety and convenience of once-weekly somapacitan in adult GH deficiency: a 26-week randomized, controlled trial
Source: Eur J Endocrinol. 2018 Feb 26;178(5):491–9. doi: 10.1530/EJE-17-1073 (PMC5920019; doi:10.1530/EJE-17-1073)

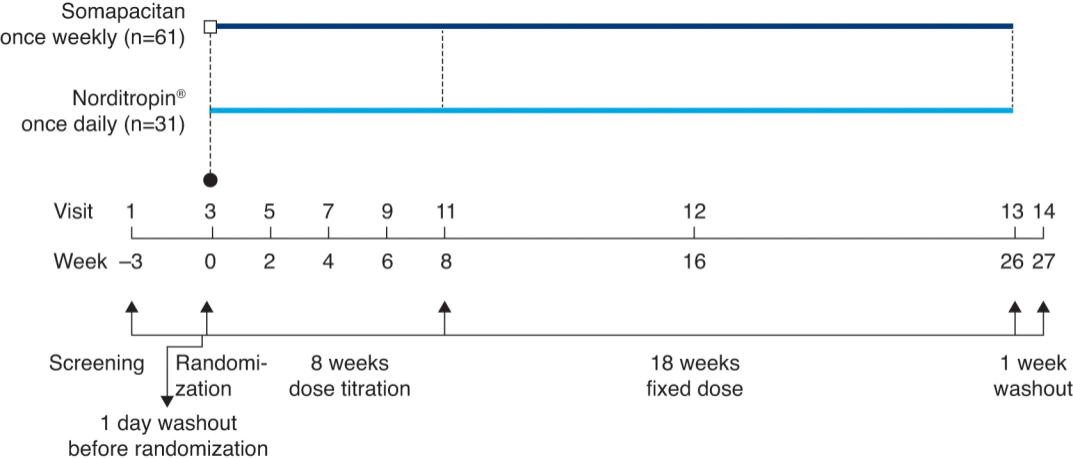

Supplement: Supporting Figure 1 [file eje-178-491-s001.pdf]
