# Supplementary material for: Safety and convenience of once-weekly somapacitan in adult GH deficiency: a 26-week randomized, controlled trial
Source: Eur J Endocrinol. 2018 Feb 26;178(5):491–9. doi: 10.1530/EJE-17-1073 (PMC5920019; doi:10.1530/EJE-17-1073)
Supplement: Supplementary material [file eje-178-491-s002.pdf]

Safety and Convenience of Once-Weekly Somapacitan in Adult GH Deficiency: A 26-Week Randomized, Controlled Trial

Gudmundur Johannsson, Ulla Feldt-Rasmussen, Ida Holme Håkonsson, Henrik Biering,

Patrice Rodien, Shigeyuki Tahara, Andrew Toogood, Michael Højby Rasmussen

Supplementary material

Details of randomization

Randomization codes were generated by the sponsor and administered centrally by an interactive web-based response system (IWRS). The randomization was stratified by region (Japan and all other countries), sex (male and female) and diabetic status (diagnosed with diabetes vs not diagnosed with diabetes). Patient randomization numbers were allocated sequentially in the order in which the patients were enrolled. Patients were enrolled by the investigators, who could access the IWRS at any time via the internet or telephone.
