# Supplementary material for: Safety and convenience of once-weekly somapacitan in adult GH deficiency: a 26-week randomized, controlled trial
Source: Eur J Endocrinol. 2018 Feb 26;178(5):491–9. doi: 10.1530/EJE-17-1073 (PMC5920019; doi:10.1530/EJE-17-1073)
Supplement: Supporting Table 1 [file eje-178-491-t001.pdf]

Supplementary Table 1. Inclusion and exclusion criteria.

Inclusion criteria:

Patients were required, at screening, to fulfill all the criteria below:

1	Informed consent obtained before any trial-related activities. Trial-related activities are any procedures that are carried out as part of the trial, including activities to determine suitability for the trial
2	Male or female of at least 18 years of age and not more than 79 years of age at the time of signing informed consent
3	GHD fulfilling either one of the following criteria:<br>a. Adult onset: subjects diagnosed with growth hormone deficiency (GHD), either alone or associated with multiple hormone deficiencies (hypopituitarism), as a result of pituitary disease, hypothalamic disease, surgery, radiation therapy, or traumatic brain injury<br>b. Childhood onset: Subjects who were GH deficient during childhood as a result of congenital, genetic, acquired, or idiopathic causes
4	Adult GHD (AGHD) diagnosed ≥ 6 months (defined as 180 days) prior to screening
5	Treatment with human GH (hGH) for at least 6 months (defined as 180 days) at screening
6	If applicable, hormone replacement therapies for any other hormone deficiencies, adequate and stable for at least 90 days prior to randomization as judged by the investigator
7	Subjects without diabetes mellitus or subjects diagnosed with diabetes mellitus (not for Japan), provided that ALL the following criteria are met:<br>diabetes mellitus (diagnosed clinically) ≥ 6 months prior to screening<br>stable oral anti-diabetic (OAD) treatment, defined as unchanged medication and unchanged dose for ≥90 days prior to screening<br>no history of use of injectable anti-diabetic agents<br>HbA_{1c} <7.0% at screening according to central laboratory<br>no diabetes-related co-morbidities (as judged by the investigator) at screening<br>fundus photography performed ≤90 days prior to randomization without proliferative retinopathy or severe non-proliferative diabetic retinopathy.

Exclusion criteria:

Eligible patients were not allowed to meet any of the exclusion criteria below:

1 Known or suspected hypersensitivity to trial product(s) or related products

2 Previous participation in this trial. Participation is defined as signed informed consent

3 Female who is pregnant, breast-feeding or intends to become pregnant or is of childbearing potential and not using adequate contraceptive methods (adequate contraceptive measures as required by local regulation or practice)

a. FOR GERMANY ONLY: Adequate contraceptive measures are implants, injectables, combined oral contraceptives, hormonal intrauterine device, sexual abstinence or vasectomized partner

b. FOR SWEDEN ONLY: Adequate contraceptive measures are:

i. oral (except low-dose gestagen (lynestrenol and norestisteron))

ii. injectable, or implanted hormonal contraceptives

iii. intrauterine device, intrauterine system (for example, progestin-releasing coil)

iv. vasectomized male (with appropriate post-vasectomy documentation of the absence of sperm in the ejaculate)

c. FOR DENMARK AND UK ONLY: Adequate contraceptive measures are intrauterine devices, hormonal contraceptives (contraceptive pills, implants, transdermal patches, hormonal vaginal devices or injections with prolonged release), or be postmenopausal defined as 12 months or more with no menses prior to enrollment

d. FOR JAPAN ONLY: Adequate contraceptive measures are abstinence, diaphragm, condom [by the partner], intrauterine device, sponge, spermicide or oral contraceptives

4 Male of reproductive age who or whose partner(s) is not using adequate contraceptive methods (adequate contraceptive measures, as required by local regulation or practice)

5 Receipt of any investigational medicinal product within 180 days before screening or participation in another trial within 90 days before randomization

6 Any disorder which, in the opinion of the investigator, might jeopardize subject’s safety or compliance with the protocol

7 Active malignant disease or history of malignancy. Exceptions to this exclusion criterion:

a. Resected *in situ* carcinoma of the cervix and squamous cell or basal cell carcinoma of the skin with complete local excision

b. Subjects with GHD attributed to treatment of intracranial malignant tumors or leukemia, provided that a recurrence-free survival period of at least 5 years is documented in the subject's medical records

8 For subjects with surgical removal or debulking of pituitary adenoma or other benign intracranial tumor within the last 5 years: Evidence of growth of pituitary adenoma or other benign intracranial tumor within the last 12 months (defined as ≤365 days) before randomization. Absence of growth must be documented by two post-surgery MRI or CT scans. The most recent MRI or CT scan must be performed ≤9 months (defined as ≤270 days) prior to randomization

9 Clinically significant hepatic disease defined as alanine aminotransferase (ALT) level greater than 3 times upper normal limit according to the central laboratory measurements

10 Clinically significant chronic renal impairment defined as creatinine level greater than 1.5 times upper normal limit according to the central laboratory measurements

11 History of positive results of tests for hepatitis B and/or C

ONLY APPLICABLE TO JAPAN: History of positive results of tests for hepatitis B and/or C or suspicion of hepatitis

12 History of positive result of test for HIV antibodies

13 Mental incapacity or language barriers which preclude adequate understanding or cooperation, who are unwilling to participate in the trial or who in the opinion of their general practitioner or the investigator should not participate in the trial

14 Female subject who plans to change estrogen therapy during the trial

15 FOR JAPAN ONLY: Diabetes mellitus (1)

Removal of patients from therapy and assessment

Treatment discontinuation criteria

1 The patient may discontinue treatment at will at any time

2 Treatment may be discontinued at the discretion of the investigator due to a safety concern or if the patient is judged non-compliant with trial procedures

	Treatment must be discontinued if the following applies:
3	Adverse event (AE): If a patient reports symptoms which are considered unacceptable by the subject or the investigator, regardless of relationship to trial product, treatment must be discontinued
4	Development of neutralizing antibodies to somapacitan defined by two consecutive samples found positive for *in vitro* neutralizing antibodies that results in clinically relevant impact on PK, PD and/or safety
5	Pregnancy
6	Intention of becoming pregnant during the trial
7	ONLY APPLICABLE TO JAPAN: If physicians definitely diagnose diabetes mellitus in accordance with diagnostic criteria established by the Japan Diabetes Society during the course of the trial the subject should be discontinued from treatment
	**Withdrawal criteria**
1	The subject may withdraw at will at any time
	The subject must be withdrawn if the following applies:
2	Included in the trial in violation of the inclusion and/or exclusion criteria
	**Patient replacement**
	Patients who discontinued treatment or were withdrawn were not replaced
