# Supplementary material for: Safety and convenience of once-weekly somapacitan in adult GH deficiency: a 26-week randomized, controlled trial
Source: Eur J Endocrinol. 2018 Feb 26;178(5):491–9. doi: 10.1530/EJE-17-1073 (PMC5920019; doi:10.1530/EJE-17-1073)
Supplement: Supporting Table 2 [file eje-178-491-t002.pdf]

Supplementary Table 2. Summary of Treatment Satisfaction Questionnaire for Medication (TSQM-9) scores by visit: full analysis set.

	**Effectiveness**		**Convenience**		**Global satisfaction**	
	**Somapacitan**	**Norditropin**	**Somapacitan**	**Norditropin**	**Somapacitan**	**Norditropin**
**FAS, N**	**61**	**31**	**61**	**31**	**61**	**31**
**Baseline**						
N	56	31	58	31	57	31
Mean (SD)	69.3 (17.7)	71.0 (21.7)	68.3 (18.3)	71.7 (17.5)	70.4 (20.1)	74.8 (18.5)
**Week 26**						
N	58	28	58	28	58	28
Mean (SD)	77.9 (15.9)	75.6 (20.1)	83.8 (12.9)	75.8 (19.1)	76.4 (19.3)	75.0 (19.8)
**Change in score (Week 26 – baseline)**						
N	53	28	55	28	54	28
Mean (SD)	9.7 (18.1)	3.8 (27.4)	15.3 (20.9)	3.0 (16.5)	5.4 (21.0)	–1.2 (15.2)

Observed values. FAS, full analysis set
